# Access to perinatal doula services in Medicaid: a case analysis of 2 states

**DOI:** 10.1093/haschl/qxae023

**Published:** 2024-03-04

**Authors:** Cara B Safon, Lois McCloskey, Maria Guadalupe Estela, Sarah H Gordon, Megan B Cole, Jack Clark

**Affiliations:** Department of Health Law, Policy, and Management, Boston University School of Public Health, Boston, MA 02118, United States; Department of Community Health Sciences, Boston University School of Public Health, Boston, MA 02118, United States; Rochester, MN 55902, United States; Department of Health Law, Policy, and Management, Boston University School of Public Health, Boston, MA 02118, United States; Department of Health Law, Policy, and Management, Boston University School of Public Health, Boston, MA 02118, United States; Department of Health Law, Policy, and Management, Boston University School of Public Health, Boston, MA 02118, United States

**Keywords:** maternal health, doula care, Medicaid

## Abstract

Doula services support maternal and child health, but few Medicaid programs reimburse for them. Through qualitative interviews with key policy informants (*n* = 20), this study explored facilitators and barriers to Medicaid reimbursement through perceptions of doula-related policies in 2 states: Oregon, where doula care is reimbursed, and Massachusetts, where reimbursement is pending. Five themes characterize the inclusion of doula services in Medicaid. In Theme 1, stakeholders recognized an imperative to expand access to doula services. Subsequent themes represent complications in accomplishing that imperative. In Theme 2, perceptions that doula services were not valued by health care providers resulted in conflict between doulas and the health care system. In Theme 3, complex billing processes created friction and impeded reimbursement. In Theme 4, internal conflict presented barriers to policymaking. In Theme 5, structural fragmentation between state government and doula communities was prominent in Massachusetts, presenting tensions during policymaking. Informants reported on problems demanding resolution to establish equitable and robust doula care policies. Medicaid coverage of doula services requires ongoing collaboration with doulas, providers, and health care advocates.

## Introduction

Maternal health indicators in the United States are highly inequitable. In 2021, the maternal mortality rate among non-Hispanic Black pregnant and postpartum people (PPP) was 2.6 times that among non-Hispanic White PPP.^1^ Severe maternal morbidity (SMM), a composite of 21 indicators reflecting end-organ dysfunction such as diabetes, high blood pressure, or infections,^2^ is prevalent among PPP, affecting over 50 000 US individuals every year^3^ and disproportionately affecting PPP of color.^4^ Evidence illuminating the positive impact of perinatal doula care on maternal health outcomes is strong and mounting. Yet, doulas are underutilized—in part, due to Medicaid reimbursement challenges.^5^ The goal of this study was to elucidate the complex process of adopting and sustaining policies covering doula care as a reimbursed health service in Medicaid.

### Doula care

Doulas are nonclinical health care personnel who provide physical and emotional support to PPP during labor and childbirth,^6^ as well as during the postpartum period. Doulas offer information about possible pregnancy-, childbirth-, or postpartum-related complications; provide continuous companionship during labor; offer physiological pain-management techniques; and provide approaches to infant care and parental coping skills.^7^ Doulas can facilitate communication between patients and maternal health providers, empowering PPP to advocate for their health care preferences.^8^

Despite the benefits, nationwide utilization is low—around 6% annually.^9,10^ While many individuals desire doula care,^11^ low usage rates may be due, in part, to high out-of-pocket costs of doula care, precluding many low-income PPP from accessing it. Low rates may also relate to a low supply of culturally congruent,^12^ community-based doula care, which is important in achieving the aforementioned positive outcomes.^13^ Low supply may be partially attributable to the fact that doulas, who are typically paid on a fee-for-service basis and lack access to the benefits that salaried employees often have, may spend up to 11 times more time with clients than other providers^14^ and are not compensated for transportation costs and time spent “on call.”^14^

### Medicaid and doula services

Medicaid is the primary payer for 42% of US births, including 65% of births among Black PPP and 59% of births among Hispanic PPP.^15^ Moreover, Medicaid enrollees are more than 80% more likely than those with private health insurance coverage to experience SMM.^3^ Including doula care as a Medicaid-covered benefit could improve access to a critical maternal care intervention.

Public and private payers infrequently reimburse for doula services.^13,16,17^ As of 2023, only 10 states reimbursed for doula services in Medicaid, and at varying rates. Challenges in state-level approaches to reimbursing for doula services remain,^18,19^ including determining doulas' scope of services (ie, identifying and defining prenatal vs postpartum vs full-spectrum doulas, as well as the extent to which doulas are perceived to express opinions or offer advice to their clients during labor and delivery and while in the presence of their provider[s]), ensuring accessibility of training, implementing standards for credentialing (ie, licensing or certification), addressing burdensome administrative requirements when enrolling as a Medicaid provider, and establishing and implementing equitable compensation rates.^13^

### Objective

A recent scoping review by our research team identified 5 key policy issues related to the implementation of Medicaid reimbursement for doula services: increasing doula workforce diversity, reducing administrative barriers to doulas' service provision, determining the value of doula services and facilitating adequate compensation, developing sustainable funding models, and strengthening partnerships between policymakers and the doula community.^20^ Resolving these issues is integral to implementing and sustaining Medicaid reimbursement for doula care but is complicated by the exigencies of policymaking in each state. To understand how the policy issues identified in the scoping review are playing out, we examined the implementation of Medicaid reimbursement for doula services in 2 states: Oregon, the state with the longest-standing Medicaid reimbursement policy, and Massachusetts, where, at the time of writing, a policy was about to be implemented. In terms of the Exploration, Preparation, Implementation, Sustainment (EPIS) framework,^21^ the states represent 2 phases of implementation: Sustainment and Preparation, respectively. The definition of EPIS phases framed the sampling and comparison of the 2 state case studies.

### Framework

In this study, EPIS defines external factors, relationships among stakeholders, and inner contextual factors that affect the implementation of social policies in complex settings, such as Medicaid, at successive stages. In the first, Exploration, multiple interested parties—state agencies, independent health policy groups, and service providers—define a new policy as they become aware of an unmet need as well as a policy option addressing it.^21^ Exploration may lead to Preparation to adopt that policy, ascertaining barriers and facilitators of implementation, assessing adaptation needs, and developing a plan to mitigate potential barriers and capitalize on potential facilitators.^21^ In Implementation, implementers discover whether efforts made during Preparation addressed potential issues; in Sustainment, the policy becomes engrained, a process that typically involves funding and ongoing monitoring and evaluation.^21^ Each phase is affected by the outer context, or policy environment; the inner context, or stakeholders' diverse interests and prior relationships; bridging factors, including the involvement of intermediary stakeholders or agreements to manage diverse interests; and innovation factors, characteristics of the policy and the policy's compatibility with established practices.

### The settings: Oregon and Massachusetts

Oregon was the first state to include doula care in Medicaid coverage, passing HB 3311 in 2011, which required the Oregon Health Authority (OHA) to consider prenatal, childbirth, and postpartum doula benefits. Thereafter, Oregon passed additional legislation to establish the Traditional Health Worker Commission, an entity that advises the OHA in matters related to the implementation of traditional health worker (THW) services as those covered by Medicaid. Traditional health workers are trusted members of their communities and are trained in at least 1 of 6 worker types, including doulas. Then, in 2014, policy implementation of doula coverage began.^22^ (Additional information on Oregon's policy is provided in Appendix S1.)

The policy in Massachusetts was in development as of November 2023. Following a succession of bills since 2018, Massachusetts' Medicaid program (MassHealth) began reimbursing for doula services in late 2023. H. 1182 was proposed in 2018, with legislation updated to H. 2372 in 2021, although that bill never moved past the House Committee on Ways and Means. A revised bill proposed in 2023 was referred to the Committee on Healthcare Financing.^23^ Around the same time, the Betsy Lehman Center for Patient Safety issued a report outlining policy change efforts to increase access to doula care.^24^ Among major findings, the report highlighted barriers to access to doula care at the client and doula levels.^24^ At the client level, there was a lack of awareness of doula services; at the doula level, doulas sought affordable training and credentialing requirements, as well as fair and equitable compensation for their services.^24^ In addition, doulas seeking to support clients faced resistance from maternity care teams and burdensome certification requirements.^24^ (Additional information is provided in Appendix S1.)

## Data and methods

### Sampling

To examine preparation for policy implementation and policy sustainment in our 2 states of interest, we conducted a qualitative study consisting of semi-structured interviews with key policy informants. We followed Kingdon’s Multiple Streams Approach,^25^ which posits that various solutions exist to resolve a given policy issue given the ambiguity of policy problems,^26^ in defining the sample of informants representing diverse perspectives and solutions in resolving policy issues. We identified informants based on their roles in the policymaking process in the 2 states and via snowball sampling, guided by informants' referrals and the primary investigator's professional connections. The sample (*n* = 20) included state legislators and representatives from state Medicaid agencies, state departments of health, and community doula organizations (Appendix S2).

### Data collection

We developed 2 semi-structured interview guides, 1 tailored to each state (Appendix S3). After the first 3 interviews, we revised the guides to improve question clarity and sharpen the interview focus. We conducted the interviews, lasting 20 to 90 minutes, between April and December 2022.

We focused on informants' perceptions of barriers and facilitators to expanded access to doula services through Medicaid. The primary investigator, who conducted all the interviews, practiced reflexivity, whereby researchers acknowledge the carrying of their own experiences, expertise, and biases to the work.^27,28^ While she identifies as a White woman without current doula training and is therefore unable to speak to the experiences of practicing or aspiring doulas of color, she is an experienced researcher on the topic of Medicaid reimbursement for doula care. After obtaining oral consent to participate and record the interviews, she conducted the interviews and transcribed transcripts using Microsoft Teams, storing data on a secure cloud server.

We received Institutional Review Board (IRB) approval from the Boston University Medical Campus IRB (H-42376). We report data in compliance with the Standards for Reporting Qualitative Research.^29^

### Analytic approach

The primary investigator and a research assistant used a combined inductive and deductive approach to identify emergent themes. Each of them open-coded 3 interviews, reflecting 10% of the sample. This enabled the development of a "coding frame", intended to capture "analytically significant features of the data".^30^ We derived an initial code list based on that subset of interviews before expanding it to generate a draft codebook. To establish intercoder reliability, each of the 2 analysts coded each of the remaining transcripts using Dedoose (SocioCultural Research Consultants),^31^ resolving discrepancies in coding applications by consensus. During this process, we also iteratively refined the codebook (Appendix S4). Next, we grouped codes by category to identify themes. We drew upon EPIS to inform the interpretation of our analysis. Finally, we member-checked^32^ with informants to ensure collaboration as we drew inferences from their responses.

## Results

**Figure 1.**
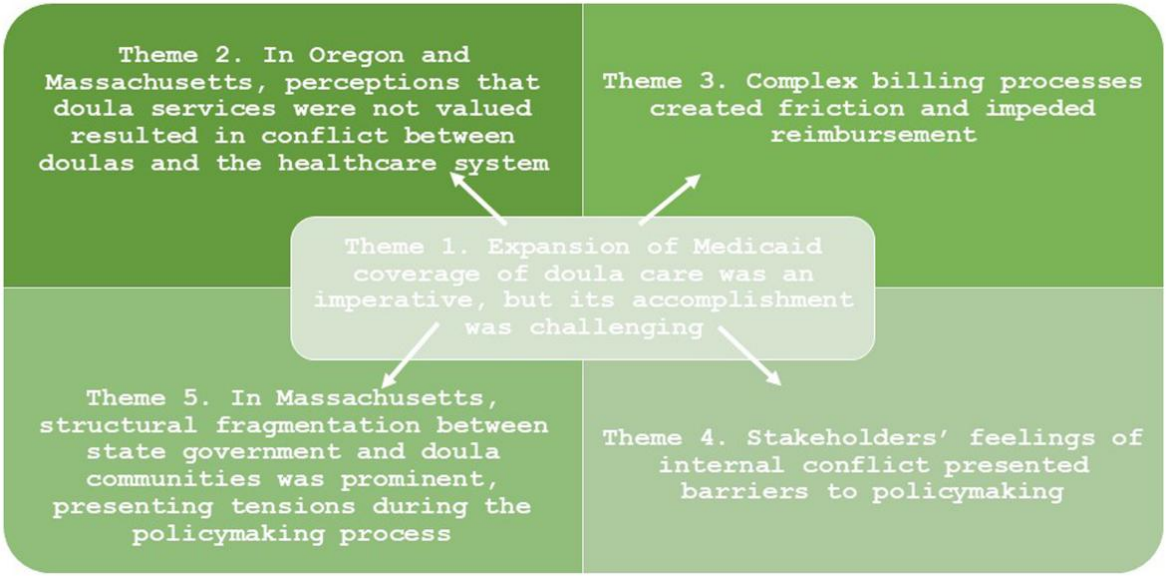
Conceptual Framework. Source: Authors.

We interviewed 8 key informants (KIs) based in Oregon and 12 in Massachusetts. Across both states, 13 were public sector informants: legislators, Medicaid agency employees, and state department of health employees; and 7 were private sector informants: local doula organization members, obstetrician-gynecologists, and employees of local health care organizations (Table 1). Five themes characterize the barriers and facilitators in policy Preparation and Sustainment (Figure 1).

**Table 1.** Informant characteristics.

Kingdon-identified sector^a 25–26^	Role	OR	MA	Total (column)
Public sector: state government (legislative branch)	Legislator	1	1	2
Public sector: state government (executive branch)	State Department of Health representative	2	3	5
Private sector: local doula organization	Local doula organization member^b^	2	3	5
Private sector: local health care organization	Local health care organization	3	3	6
Private sector: clinicians/physicians	Obstetrician-gynecologists	0	2	2
Total		8	12	20

Abbreviations: MA, Massachusetts; OR, Oregon.

Source: Authors.

^a^Informants categorized according to Kingdon-identified sector may also identify as doulas and/or as doula care advocates.

^b^In Oregon, may include local billing hubs, Independent Physician Associations (IPAs), health care providing organizations, and local Coordinated Care Organizations (CCOs) in OR, or Accountable Care Organizations (ACOs) in MA.

### Theme 1. Expansion of Medicaid coverage of doula care was an imperative, but its accomplishment was challenging

Inherent in the fact that Oregon had pursued and Massachusetts was in the midst of pursuing Medicaid reimbursement for doula care, public insurance coverage of doula services was a critical goal for all stakeholders. Yet, while stakeholders agreed about the need to include doula services as a covered Medicaid benefit, Oregon-based informants reported issues with both doula care service implementation and Medicaid policy sustainment. One informant noted challenges in identifying fair and equitable reimbursement rates for doulas, lamenting, “it’s hard to be a leader when you don’t have someone to look up to sometimes” (KI 7—state government, Oregon).

In Massachusetts, informants were challenged with pressures to create comprehensive doula care policy. One informant described the low likelihood of including full-spectrum doula care—care provided prenatally, during labor and delivery, and postpartum, as well as for pregnancies that do not result in live births—as a MassHealth-covered benefit from the outset, implying that it would take time to solidify the policy to make it inclusive of all pregnancy outcomes. She said, “Sometimes it’s easier to kind of get an imperfect policy through the door first and build onto it afterwards” (KI 6—state government, Massachusetts).

Another stakeholder noted the nuances of developing an appropriate credentialing system for doulas when no roadmap detailing the process exists: “there’s not much of a blueprint” so developing “a doula initiative and a reimbursement pathway…it's…a beast of a thing” (KI 8— state government, Massachusetts).

### Theme 2. In Oregon and Massachusetts, perceptions that doula services were not valued resulted in conflict between doulas and the health care system, specifically related to doulas’ ability to attend births

Informants felt that clinicians often overlooked the value of doula services. This was evident in reports of doulas' inability to access maternity care settings. Where hospital policies restricted nonclinical personnel from the delivery or operating room, doulas were often excluded, even when clients expected their doulas' presence. Members of doula services–providing organizations, among others, reported a need for strategies to manage conflicts that often emerged when clinicians and health care organizations believed that doulas were ignoring the patient. One Massachusetts-based informant said:

I hear a lot of the gripes from the nurses about the doulas. Yeah, those doulas show up, and they just sit in the corner and they don’t do anything or, you know, like they’re worthless…just sitting there doing nothing. (KI 5—local doula organization, Massachusetts)

On the other hand, doulas were often perceived to overstep boundaries, such as offering medical advice. The same Massachusetts informant reported:

We are not your enemy here…don’t kick us out of the room during the epidural. Don’t kick us out of the room and from the OR…Don’t kick us out at the most critical moments.

An Oregon doula echoed the sense of injustice of being “kicked out” of the delivery room:

I’m being very extra mindful of my role [in the hospital] and how I’m interacting with people because I’m like, I don’t want, you know, the provider to feel uncomfortable and be like, ‘why are you saying these things now?’ You can’t be like kicking me out of the room. (KI 1—local doula organization, Oregon)

In addition, emergent circumstances sometimes prevented the doula from attending a birth, such as when a client delivered more quickly than expected, delivered outside the hospital, or experienced a medical emergency, which prevented the doula’s presence. Doulas' absence from their clients' labor and deliveries could result in non-compensation. Informants felt this was unfair, prompting a call for better policy:

…that is not fair that it’s the doula who’s carrying the burden of that loss [of compensation] exclusively. That loss needs to be shared by the hospital, by MassHealth…[the loss] needs to be somehow equally divided so that it’s not just the doula who’s responsible to pay for her training…not the doula who’s trying to make a living by doing her best effort to show up. (KI 5—local doula organization, Massachusetts)

Relatedly, many informants believed that the earlier in the prenatal period that clients and doulas engaged, the better. However, they noted that state policies often limited the number of prenatal and postpartum visits covered by Medicaid. One informant in Massachusetts reflected that limited prenatal interactions in contracts between doula services organizations and Medicaid Accountable Care Organizations (ACOs) might inhibit the establishment of doula–client rapport and trust, reducing the chances of the doula being called to the birth:

[Earlier doula-client engagement]…leads to the likelihood that that client will call their doula when the time comes to go to the hospital. However, you know there is a ceiling to the number of allowable visits in most contracts, so…the doula needs to find a way to keep the momentum going outside of those visits. (KI 7—local doula organization, Massachusetts)

Finally, some informants argued that the state should play a stronger role in preparing for doulas' integration into the health care system. One informant in Massachusetts suggested that the state create policies promoting better communication between doulas and providers:

I think the state is going to have to take on some responsibility for making sure that those hospitals and providers, not only are, you know, welcoming of doulas but are prepared, in terms of like having consistent policies and, you know, having infrastructure for feedback loops both from the doulas and the providers. (KI 1—local health care organization, Massachusetts)

### Theme 3. Complex billing processes created friction and impeded reimbursement

Billing Medicaid for doula services and receiving payment presented challenges in both states. One informant involved in billing in Oregon described the experience of a doula living in a rural community who struggled, due to geographic and financial barriers, to bill for her services according to the requirement that bills be submitted by fax:

…requiring a form be faxed is creating barriers so and then of course, you know, not only supporting the doula and figuring out where can she go to find a fax machine…but also, and by the way, she can’t afford the FedEx, you know, going to a FedEx Office. It’s like $2.50 a page and it’s like 15 pages. (KI 5—local health care organization, Oregon)

The same informant suggested that doulas with practical billing experience might be burdened with requests for “free” support from their peers (KI 5—private sector employee, local health care organization, Oregon).

Finally, 1 informant in Massachusetts lamented that, because doulas were viewed as external to the health care system, they lacked the billing departments or outsourced arrangements that often supported the billing process from which physicians often benefitted. This left some doulas not knowing about which of their services might be billable to MassHealth:

…a doctor…has a 5-minute phone call with somebody about a prescription refill. You better believe that there’s a billing specialist somewhere in his department that’s gonna bill for that. But right now, because doulas are operating outside of the medical system…nobody’s looking over their shoulder to flag things that are billable. (KI 7—local doula organization, Massachusetts)

Even when billing issues were managed well, some informants in Oregon reported challenges in ensuring doulas' reimbursement from insurance carriers contracted with Medicaid Coordinated Care Organizations (CCOs). From the perspective of 1 business owner, there were problems with obtaining reimbursement for doulas:

I’m the one calling them and kind of nagging them like, ‘hey, where’s our payment or what happened here?’… I should not be having to prove that this is the rate that we agreed, you know, for you guys to pay. (KI 1—local doula organization, Oregon)

Informants also reflected on problems with reimbursement structures. One informant noted that doulas were often paid the same amount regardless of the length of clients' labor and delivery, and that doulas were often not paid for all services:

…you never know if [the birth is] gonna be 3 hours or 20 hours….I think that there should be an hourly rate for every hour worked and that includes phone time, text time, travel time, some compensation for being on call that's the time either virtual or in person. (KI 5—local doula organization, Massachusetts)

Another informant from Oregon contrasted private pay doulas, who are paid up front by their clients, with Oregon Health Plan (OHP) (Appendix S1) doulas. If the latter were not contracted with CCOs, they needed to wait until all services were rendered to receive reimbursement in a timely manner. The delay reflected poorly on organizations providing doula services and dissuaded doulas from serving OHP clients:

…it makes us look bad as an organization that we’re not paying people and so that discourages doulas…to work with OHP patients or clients. (KI 6—local doula organization, Oregon)

### Theme 4. Stakeholders' feelings of internal conflict presented barriers to policymaking

Informants told of conflicts between candor with doulas and the absence of transparency that can characterize political discourse. The conflict was acute for those who were both practicing doulas or doula advocates and practicing policymakers. One informant described the intricacies involved in navigating a given political situation:

I constantly felt like I was walking a tightrope of not being able to be transparent with doulas because of confidentiality reasons, and then feeling like I had to protect doulas when I was advocating for the policy. (KI 6—state government, Massachusetts)

Another informant illustrated the tension between personal values and exigencies of policymaking as she dealt with differences between the number of prenatal and postpartum visits that met the needs of doula clients, and what was considered feasible. As a public sector employee and a “practicing doula,” her state role required a calculation of the reimbursement rate for what she considered to be the unquantifiable value of patient-centered doula services:

Financially, in terms of structures and support, I understand professionally why [the stipulated number of prenatal and postpartum visits] are what they are…as a practicing doula, as someone who has…been to over 100 births…I think that there’s no possible way to put a quantitative number to that support. (KI 8—state government, Massachusetts)

While most informants agreed that the then-anticipated 2022 reimbursement rate increase from $750 to $1500 in Oregon (Appendix S1) would benefit the doula community and their clients, some informants expected that the increase would attract doulas unaccustomed to serving low-income PPP, and thus “breed a workforce” of doulas used to serving wealthy clients who paid out of pocket for doula services. One informant looked forward to the expansion of the doula workforce while expressing concern about those doulas' having sufficient training and education about resources for OHP clients:

[The rate increase] is going to bring people who were mostly serving private pay clients into the Medicaid space…and I just think that that could breed a workforce that is more adept at serving a higher income population. And so do they understand basic things like where food banks are and the needs of folks who don’t have transportation? (KI 5—local doula organization, Oregon).

Another informant, from Massachusetts, worried about the administrative requirements of Medicaid burdening doulas. She argued that, instead of a Medicaid-only approach, additional funding streams were needed both to reduce the burden for doulas and to expand access to services:

…in my ideal world, there would be more paths to covering doula services through like foundations or through grant-based programs that have less of these, like, hoops you have to jump through as a provider and so I just worry that there’s like so much focus on Medicaid, and I don’t know if Medicaid is the right answer for improving perinatal health and increasing access to doula services. I think we’ve seen in a lot of states it kind of does the opposite. (KI 6—state government, Massachusetts)

Internal conflict also emerged as a concern between doulas. One private-sector employee with previous doula training and experience noted this disagreement “across doulas” in the state, highlighting that some doulas wished to be viewed as part of maternity care teams, while others valued their role outside of it:

I’ve definitely learned…what kinds of concerns doulas tend to have, especially when you talk about integrating doula care into the health care system or into these larger institutions, which is not the way the traditional doula role has looked. And there’s not always agreement across doulas in terms of…what this should look like. (KI 1—local health care organization, Massachusetts)

Despite this, many informants shared a desire to increase and maintain alignment among the doula community. Noting issues with identifying as a doula when doula care might not be uniformly defined—let alone uniformly known among health care providers who engage with doulas—1 informant expressed the opinion that doulas sharing a collective identity would be advantageous. This was despite the fact that doulas were believed to hold different opinions about the extent to which they wished to be integrated into maternity care teams:

…there’s a huge spectrum of how people look at themselves as a doula. It's hard for other health professionals to understand what that doula is if they can’t come together and define that themselves. (KI 3—state government, Massachusetts)

### Theme 5. In Massachusetts, structural fragmentation between state government and doula communities was prominent, presenting tensions during the policymaking process

In Massachusetts, lack of cohesion within and between groups of stakeholders, such as within the state and among doulas, as well as between the state and doulas, was present. This often bred misinformation, misunderstanding, or misalignment of operations between groups of stakeholders.

Informants described fragmentation between federal- and state-level requirements, leading to knowledge about policy among some but not others that exacerbated existing fragmentation:

…most [legislators] have never worked in government before, they’ve never designed programs or policies directly before. So they just don’t understand the level of detail needed to think through these things. (KI 6—state government, Massachusetts)

Where policymakers did not understand how Medicaid operated, some informants elaborated on the challenges in creating an equitable policy that functioned well for both Medicaid and doulas. Each of the state agencies at play came with its own interests and agendas. One state legislator said:

…the way state government works is often very siloed…we’ve tried to pull together meetings and get everybody in the room and kind of say, ‘all right, so our end goal is the same. So how do we make sure that the legislative branch and the executive branch are working together and we’re keeping each other in the loop about what we’re doing?’ (KI 2—state government, Massachusetts)

Structural fragmentation also manifested as a point of tension between doulas and policymakers. According to some informants, some doulas misunderstood “the legislative process,” including the extent to which state administrative agencies are permitted to engage with legislators. One informant described a lack of engagement between the executive and legislative branches, noting that doulas and advocates might not be educated about the legislative process, leading to misconceptions among those groups (KI 3—state government, Massachusetts). On the other hand, some informants believed that policymakers' lack of insight into doula workforce issues threatened the equity and robustness of policymaking, with some relating a lack of partnership between state government and the doula community. This also highlighted perceived misconceptions among doulas about what the state was permitted to do, as well as its capacity and funding. However, even when doulas acknowledged the absence of funding for the expansion of doula care, informants described a need for that funding to invest in doula workforce development and communicate effectively:

Zero dollars have been allocated to doula training and workforce development. Everybody wants the perfect outcome. But where’s the money to invest in actually getting it right? (KI 5—local health care organization, Massachusetts)

Another informant believed that certain requirements embedded in some states' policies, such as the requirement for doulas to pass an exam before becoming certified, were unnecessary. Identifying weaknesses in another state's legislation that failed to pass, which she felt was due to the exclusion of doulas' perspectives and experiences from the policymaking process, she expressed pride in her own state for having involved doulas in policymaking from the beginning (KI 1—local health care organization, Massachusetts).

As an extension of the idea that regulating doula work by establishing certification pathways might be detrimental to the workforce, some informants expressed concern that requiring certification could mean that large organizations such as DONA International, a leading training entity for doulas,^33^ would continue to be the primary training entity for doulas, while pushing out community-based organizations and precluding many doulas from diverse backgrounds from obtaining the training required for workforce entry. Many believed this to be the case due to the high costs of training from such large organizations relative to smaller, community-based ones. Informants believed that racial diversity among doulas was needed to facilitate cultural congruency, which was seen as necessary to yield identity-sharing and strong relationships between doulas and clients:

I think a big part of feeling comfortable [as a client]…is to have congruent identities with [doulas]…and I worry about creating a doula workforce that's predominantly White women who have the money to do a DONA training but have never been on Medicaid themselves. (KI 6—state government, Massachusetts)

## Discussion

We explored challenges and facilitators of Preparing to implement policy and Sustaining policy related to Medicaid coverage of doula services in 2 states that were at different stages of policymaking as characterized by EPIS. We identified 5 themes. First, underpinning all other themes, stakeholders in Oregon (Sustainment) and Massachusetts (Preparation) sought expanded access to doula care, although achieving that expansion presenting many challenges (Theme 1). Themes 2–5 reflect those challenges. Those issues related to the value of doula services as determined by providers and other health care professionals already integrated into maternity care teams (Theme 2) and the bureaucratic exclusion of doulas via complex and costly billing and reimbursement processes (Theme 3). Internal conflict also arose among various stakeholders: as informants reported, policy preparation and sustainment involved tradeoffs between what informants considered ideal (ie, coverage of unlimited prenatal and postpartum visits by doulas) vs practical (ie, caps on those visits) (Theme 4). Finally, structural fragmentation within and between informants in Massachusetts presented tensions that stifled policymaking (Theme 5). Because that fragmentation, manifested by a lack of uniformity as a doula workforce, likely stymied recognition of doulas' value, it may have also stymied their political effectiveness. The EPIS framework's Bridging factors, discussed further below, serve as one way to reduce such fragmentation.

In Oregon, sustaining reimbursement entailed establishing mechanisms for doulas to be paid, but doulas were disadvantaged by complex bureaucratic procedures and their lack of resources to manage them, such as fax machines and billing departments. In Massachusetts, doulas faced conflicts with clinicians in labor and delivery rooms, where doulas saw their own work as acutely valuable. In Massachusetts, preparing also entailed resolving barely tractable problems of the value of doula services. In both states, these problems can be characterized as issues among players both within and between EPIS' inner and outer context, as described by a table outlining EPIS constructs and domains as depicted by the cases of Oregon (Sustainment phase) and Massachusetts (Preparation phase) (Appendix S5). Conflict emerged in integrating doulas into maternity care teams (the inner context), and was shaped by factors like the service environment (eg, stakeholders' perceptions of doula care), and billing and reimbursement processes designed for established providers, not doulas (the outer context).

These issues highlight the significance of EPIS' Bridging factors: arrangements for resolving the conflicts inherent in the adoption of a policy innovation. They represent an investment in partnerships among policymakers, between policymakers and doulas, and between doulas and doula care advocates.^5,8,13,18,20,22,34-40^ In Massachusetts, the Department of Public Health's Doula Partner Advisory Group convenes monthly to discuss ongoing legislative efforts, as well as doula–health care facility partnership and doula workforce strengthening. California stakeholders held similar meetings prior to implementation of their state's policy, and continue to hold their legislatively required Doula Implementation Workgroup.^41^ Such meetings in these and other states, as supported by a "stakeholder-engaged process" in California,^42^ provide opportunities to develop the “blueprint” for doula policy deemed necessary by an Oregon-based informant to refine implementation and sustain policy.

Yet, as we found, refinement of the blueprint does not end with Preparation: Sustainment inevitably involves ongoing negotiations. In Oregon, THW liaisons, who assist doulas with submitting Medicaid claims and billing support on behalf of CCOs,^43^ have been deployed to facilitate partnership both among and between players in the inner context of service delivery and its outer context. In other states considering adoption of doula care policy, entities such as the Doula Network, a national program providing billing and other support to doulas, can unite health plans, providers, and community members^44^ to enable consensus-building. Prior research using EPIS to explain policy processes demonstrates how Bridging factors to facilitate facets of the reimbursement process are needed.^45,46^

The significance of bridges is prominent in promoting doula workforce diversity. Informants believed that racial diversity was a necessary component of maternal empowerment, particularly for PPP of color.^47^ It is vital that policies stipulate a focus on recruiting, training, and retaining a diverse workforce to address strengthen the broken maternity care system.

### Limitations

We examined policies in only 2 states, limiting our ability to draw conclusions transferrable to others. However, our findings are consistent with the research and policy literature and current national debates surrounding the integration of doula care in health care.^20^ States may refer to existing resources^48^ as they consider the various components of state support for community doula services. In addition, in an effort to focus this study on key policy informant perspectives, we did not interview PPP and birthing families; their perspective and partnership in any state's doula care policymaking process are urgently needed.

### Implications

State Medicaid policies around doula care may complement other policies that benefit PPP. The American Rescue Plan Act of 2021 (ARPA) offers states the option to extend postpartum coverage of Medicaid from 60 to 365 days. Expanding Medicaid eligibility to nonelderly adults, as enabled under the Affordable Care Act, may result in more PPP being enrolled and engaged in care earlier, and therefore, more likely to utilize doula services. In states with the postpartum ARPA policy,^49^ which offers PPP 365 instead of 60 days to take advantage of the benefits of Medicaid enrollment during the postpartum period, the extension of benefits means that Medicaid-enrolled PPP could have more time to take advantage of the doula care benefit in the postpartum period. State decisions to expand postpartum Medicaid eligibility could motivate those states to expand access to postpartum doula services in Medicaid and in commercial insurance carriers, especially given recent findings about the health benefits and cost savings of doula care.^50^

## Conclusion

Despite stakeholders sharing a policy agenda, doula care policymaking generated conflict during policy sustainment in Oregon and in policy implementation in Massachusetts. We found that, consistent with extant literature on doula care coverage for doula services, regardless of policy adoption stage, developing equitable and robust doula care requires collaboration with doulas, providers, and maternal health care advocates.

## Supplementary Material

qxae023_Supplementary_Data
