# Supplementary material for: Access to perinatal doula services in Medicaid: a case analysis of 2 states
Source: Health Aff Sch. 2024 Mar 4;2(3):qxae023. doi: 10.1093/haschl/qxae023 (PMC10986220; doi:10.1093/haschl/qxae023)
Supplement: qxae023_Supplementary_Data [file qxae023_supplementary_data.zip › Appendix A1_Supplemental Material-Case Studies 1 and 2_R-R.docx]

**Case Study 1: State-level Medicaid Reimbursement for Doula Services in Oregon**

Per early legislative language, doulas must operate under the supervision of a licensed healthcare practitioner and enroll in the state registry as THWs. As specified in a State Plan Amendment (SPA) introduced in in 2017 and approved in July of that year, the state increased the reimbursement rate from its original level of $75 for attendance at labor and delivery only to $350, reflecting $50 for each of two prenatal and two postpartum visits, as well as labor and delivery for $150.^1^ That SPA also removed the requirement for doulas to obtain a referral from other licensed obstetric birth providers.^1^ Some doulas successfully negotiated higher rates, and some coordinated care organizations (CCOs) – networks of healthcare providers similar to accountable care organizations (ACOs) but whose governance structures rely on employing patient-centered medical homes, global budgets, and community advisory panels^2, 3^ serving individuals in their local communities in the state’s Medicaid program – the Oregon Health Plan (OHP)^4^ – often paid higher rates than these determined fee-for-service rates.^5^ In June 2022, the OHA announced plans to submit a new SPA to CMS to increase the reimbursement rate to $1,500 per pregnancy, inclusive of at least two prenatal visits and two postpartum home visits.^6^ CMS approved the SPA with an effective date of July 1, 2022.^7^

In spite of this progress for the doula workforce, OHP only reimbursed doulas for their services relative to 204 births between 2015 and 2020, even though about 19,000 Medicaid enrollees give birth annually in Oregon.^8^ The literature highlights challenges inherent in the billing and reimbursement process for doulas in Oregon; as such, in 2017, CMS approved the state’s request to remove the requirement for clients to be referred to a doula by a licensed provider.^9^

As of October 1, 2022, to gain certification, doulas must complete 40 hours of OHA-approved trainings, including 28 in-person contact hours of training in cultural competency, inter-professional collaboration, HIPAA compliance, and trauma-informed care.^10^ Their core curriculum must cover a slew of content areas, including anatomy and physiology of labor, birth, postpartum, neonatal transition, and breastfeeding.^10^ After that, they must become a certified and registered THW; become an Oregon Medicaid provider, first obtaining a National Provider Identifier (NPI) number; and utilize the claims format to bill for services.^10^ Oregon’s model also includes doula hubs, which enlist THW liaisons to help connect doulas with CCOs as well as offer support with OHP claims and billing.^2^

**Case Study 2: State-level Medicaid Reimbursement for Doula Services in Massachusetts**

The Massachusetts Department of Public Health (MDPH) established a Doula Initiative to build awareness of doula services throughout the state, and to build a policy alongside doulas and doula advocates as DPH seeks to gain authority to certify doulas, according to the extent to which doulas seeking certification obtain necessary training and educational requirements.^11^ The Doula Initiative seeks to support and expand a strong doula-led workforce through steps th include developing a certification pathway; enhancing ongoing workforce development opportunities; and establishing a pathway to sustainable financing.^11^ In addition, this initiative seeks to ensure high-quality doula services for Massachusetts families and raise the profile of doula work across the Commonwealth of Massachusetts^12^. The members of the Massachusetts Doula Coalition and other advocacy groups in the state advised MassHealth and DPH on policy development efforts, andwrote a new doula bill filed in 2023.^13^ The bill would enable doulas to serve pregnant and postpartum mothers up to 12 months following the end of pregnancy, including continuous support during labor and delivery, and up to six doula visits across the prenatal and one-year-postpartum period.^13^ It would also require the appointment of a doula advisory committee composed of doulas and MassHealth members, as well as a doula workforce development trust fund to expand access to doula trainings and mentorship opportunities.^13^

Separate from the legislation, MassHealth began reimbursing for doula services^14^ by doulas who enroll as Medicaid providers, releasing proposed program^15^ and rate^16^ regulations detailing its policy. Final program^17^ and rate^18^ regulations were released in December 2023, and stipulated that doulas could bill for $100 for visits up to 60 minutes, $150 for those 61-90 minutes, and $900 for labor and delivery support – regardless of pregnancy outcome.

**REFERENCES**

1. Medicaid.gov, Oregon State Plan Amendment (SPA) Transmittal Number 17-0006, C.f.M.a.M. Services, Editor. <https://www.medicaid.gov/sites/default/files/State-resource-center/Medicaid-State-Plan-Amendments/Downloads/OR/OR-17-0006.pdf>: Department of Health & Human Services, Centers for Medicare & Medicaid Services, Seattle Regional Office.

2. Crane, C, Increasing Access to Doulas in Oregon: A Delphi Study, in Health Systems & Policy. 2023, Portland State University: Portland, OR.

3. McConnell, KJ, AM Chang, DJ Cohen, N Wallace, ME Chernew, G Kautz, et al., Oregon's Medicaid Transformation: An Innovative Approach To Holding A Health System Accountable For Spending Growth*.* Healthc (Amst), 2014. 2(3): p. 163-167.

4. Oregon Health Authority: Oregon Health Plan. Coordinated Care Organizations (CCOs). No date [cited 2023 February 16]; Available from: <https://www.oregon.gov/oha/hsd/ohp/pages/coordinated-care-organizations.aspx>.

5. Catlin, D. Guidelines for THW Doulas Serving OHP Members. No date [cited 2023 February 16]; Available from: <https://www.oregon.gov/oha/OEI/THW%20Documents/Guidelines%20for%20THW%20Doulas%20Serving%20OHP%20Members.pdf>.

6. Oregon Health Authority, Public notice, in Notice of intnet - OHA will amend the Medicaid State Plan to increase fee-for-srevice reimbursement for doula services. 2022, OHA: Salem, Oregon.

7. Centers for Medicare & Medicaid Services: Center for Medicaid and CHIP Services, OR State Plan Amendment (SPA) #: 22-0019, in 22-0019, C.f.M.M. Services, Editor. 2022, Centers for Medicare & Medicaid Services: Oregon.

8. National Health Law Program. Doula Series Footnotes. 2022 [cited 2023 February 16]; Available from: <https://doulaseriesfootnotes.com/oregon-data.html>.

9. Centers for Medicare & Medicaid Services: Center for Medicaid and CHIP Services, Oregon State Plan Amendment (SPA): # 17-0006, in 17-0006, C.f.M.M.S.C.f.M.a.C. Services, Editor. 2017: Oregon.

10. Oregon Secretary of State, Oregon Health Authority Health Systems Division: Medical Assistance Programs - Chapter 410, Division 180, Traditional Health Workers, in 410-180-0375. 2022: Oregon.

11. Mass.gov. Doula Initiative. 2023 [cited 2023 September 19]; Available from: <https://www.mass.gov/info-details/doula-initiative#doula-initiative-team->.

12. Betsy Lehman Center for Patient Safety, Expanding doula support services in Massachusetts: Considerations for Successful Implementation. 2022: Boston, Massachusetts.

13. An Act Relative to Birthing Justice in the Commonwealth, in House Docket, No. 2452. 2023.

14. Reale, H. Massachusetts' new hope in fighting the maternal health crisis? Doulas. 2023 August 30, 2023 [cited 2023 September 19]; Available from: <https://www.wgbh.org/news/health/2023-08-30/massachusetts-new-hope-in-fighting-the-maternal-health-crisis-doulas>

15. Executive Office of Health and Human Services (EOHHS), Program Regulations: 130 CMR 463.000: Doula Services, C.o.M.M.P.M. Series, Editor. 2023, EOHHS: Boston, MA.

16. Executive Office of Health and Human Services (EOHHS), 101 CMR: Rates for Doula Services. 2023, EOHHS: Boston, MA.

17. Commonwealth of Massachusetts. 130 CMR 463.000 Doula Services Manual. Commonwealth of Massachusetts MassHealth Provider Manual Series [Regulation] 2023 December 8, 2023 [cited 2023 December 8]; Available from: <https://www.mass.gov/doc/doula-services-regulation-effective-december-8-2023/download>.

18. Mass Register Executive Office of Health and Human Services. 101 CMR 319.00: Rates for Doula Services. 2023 December 8, 2023 [cited 2023 December 8]; Available from: <https://www.mass.gov/doc/rates-for-doula-services-effective-december-8-2023-0/download>.
