# Supplementary material for: Access to perinatal doula services in Medicaid: a case analysis of 2 states
Source: Health Aff Sch. 2024 Mar 4;2(3):qxae023. doi: 10.1093/haschl/qxae023 (PMC10986220; doi:10.1093/haschl/qxae023)
Supplement: qxae023_Supplementary_Data [file qxae023_supplementary_data.zip › Appendix A2_Supplemental Material-Kingdon.docx]

| *KINGDON IDENTIFIED SECTOR | ROLE |
| --- | --- |
| Public sector: state government (legislative branch) | Set policy agendas |
| Public Sector: state government (executive branch) | Implement policy agendas |
| Private sector: local doula organization member | Apply resources to influence policymakers |
| Private sector: clinicians/physicians | Apply clinical skills to practice; utilize research and data |

*Informants categorized according to Kingdon Identified Sector may also identify as doulas and/or as doula care advocates.
