# Supplementary material for: Access to perinatal doula services in Medicaid: a case analysis of 2 states
Source: Health Aff Sch. 2024 Mar 4;2(3):qxae023. doi: 10.1093/haschl/qxae023 (PMC10986220; doi:10.1093/haschl/qxae023)
Supplement: qxae023_Supplementary_Data [file qxae023_supplementary_data.zip › Appendix A3_Supplemental Material_Interview Guides.docx]

***INTRODUCTION***

*Thank you for agreeing to participate in this interview. I am a PhD candidate studying access to doula care as part of the Health Services Research program at Boston University School of Public Health. As part of my dissertation research, I am conducting key informant interviews with stakeholders from Oregon (whose state Medicaid program reimburses for doula services) and Massachusetts (whose program does not) who have insight access to doula services in Medicaid.*

*The purpose of the interview is to learn your thoughts on policies to make doula care accessible to low-income populations. Your professional perspective may help inform doula care policy.*

*Some of what I state below reflects what you may have read in the Research/Exempt Information Sheet you were provided.*

- *Upon your oral consent to participate, we will be recording the interview in order to capture your responses accurately and comprehensively. If you consent to be recorded, we will also be taking field notes throughout; if you do not consent to the recording, we will take written notes only.*
- *There are no right or wrong answers to any of the questions. We encourage you to ask clarifying questions as needed throughout. As all questions posed during the interview are optional, you may skip any given question or request to stop the interview at any point.*
- *We will not compensate interviewees for participating in this study.*

*What questions can I answer for you before we begin?*

***[Start recording]***

*Today’s date is [DATE] and [TIME]. I am interviewer [NAME] and this is Interview Participant Number [#].*

**OREGON**

**INTERVIEW QUESTIONS**

**PROFESSION.**

***First, I’d like begin the interview by asking some background questions about your work.***

1. **Please tell me, briefly, about your role at the organization at which you work.**
   1. **Probe:** Please describe your experience working with compensation and reimbursement for doula services in OR.

**ACCESS TO DOULA CARE.**

***Now I would like to talk about access to doula care in Medicaid.***

1. **How do Oregon Health Plan (OHP) members come to access doula care?**
   1. **Probe:**
      - Prenatally?
      - During labor and delivery (i.e., once admitted to the hospital (for hospital deliveries))?
      - Postpartum?
   2. **Probe:** To your knowledge, how do OHP-insured individuals become aware of doula services?
   3. **Probe:** How can individuals in OHP best be informed about and gain access to doula care?
   4. **Probe:** Please describe outreach and education efforts (if applicable).
2. **From your perspective, what are some of the key reasons OHP members need doula care?**
   1. **Probe:** What are some of the barriers facing clients who seek doula care:
      - **Probe:** Prenatally?
        1. **Probe:** lack of client awareness, costs involved, geographical barriers
      - **Probe:** During childbirth/labor and delivery?
        1. **Probe:** lack of client awareness, costs involved, geographical barriers
      - **Probe:** Postpartum?
        1. **Probe:** lack of client awareness, costs involved, geographical barriers
3. **From your perspective, what are some of the key reasons OHP members want doula care?**
   1. **Probe:** What are some of the barriers facing clients who seek doula care:
      - **Probe:** Prenatally?
        1. **Probe:** lack of client awareness, costs involved, geographical barriers
      - **Probe:** During childbirth/labor and delivery?
        1. **Probe:** lack of client awareness, costs involved, geographical barriers
      - **Probe:** Postpartum?
        1. **Probe:** lack of client awareness, costs involved, geographical barriers
4. **Much attention is being paid to racial and ethnic inequities in maternal health outcomes. What role, if any, do doulas play in addressing these disparities?**
5. **[If applicable]: What are your thoughts about timing of enrollment in OHP and its relationship to OHP coverage of the doula benefit?**
   1. **Probe:** To your knowledge, what proportion of OHP-eligible individuals enroll in coverage at some point during the perinatal period?
   2. **Probe:** To your knowledge, when during the perinatal period do OHP-eligible individuals typically enroll in coverage?
6. **[If applicable]: To your knowledge, what proportion of OHP-insured individuals access doula services?**
   1. **Probe:** Prenatally?
      - **Probe:** When?
   2. **Probe:** During labor and delivery (i.e., once admitted to the hospital (for hospital deliveries))?
   3. **Probe:** Postpartum?
   4. **Probe:** When?
7. **[If time]: Please describe how OHP reimbursement for doula services came to be in 2013.**
   1. **Probe:**
      - Adoption? Please describe any issues then and now.
   2. **Probe:** HB 3311 (2011) 🡪 HB 3650 🡪 SB 1580
   3. **Probe:** Development of the Oregon Doula Association (ODA)
   4. **Probe:** SPA (2017)
   5. **Probe:**
      - Implementation? Please describe any issues then and now.
8. **[If time]: How is OHP reimbursement for doula care currently configured in OR?**
   1. **Probe:** Please speak to issues related to doulas’ scope of services.
   2. **Probe:** “ “ doula training and core competencies.
   3. **Probe:** “ “ licensure and certification.
   4. **Probe:** “ “ relationship to clinical providers.
   5. **Probe:** “ “ process of compensation and reimbursement process.
   6. **Probe:** “ “ quality assurance and supervision.
9. **How do you feel about OR’s policy to increase access to doula care for OHP members (i.e., from your perspective, what value do doulas bring)?**

**THE FUTURE OF DOULA CARE.**

- - - 1. **In your opinion, what do you think should be done, if anything, at the national level with respect to supporting access to doula care?**

**CLOSING.**

- - - 1. **Would you like to share anything else that we have not covered during this interview?**

Those are all the questions I have for you at this time. Thank you again for your participation.

**[Stop recording]**

**MASSACHUSETTS**

**INTERVIEW QUESTIONS**

**PROFESSION.**

***First, I’d like begin the interview by asking a background question about your work.***

1. **Please tell me, briefly, about your role at the organization at which you work.**
   1. **Probe:** Please describe your experience working with issues related to compensation and reimbursement for doula services in MA.

**ACCESS TO DOULA CARE.**

***Now I would like to talk about access to doula care in Medicaid.***

1. **From your perspective, what are some of the key reasons MassHealth-eligible people need doula care?**
   1. **Probe:** What are some of the barriers facing clients who seek doula care:
      - **Probe:** Prenatally?
        1. **Probe:** lack of client awareness, costs involved, geographical barriers
      - **Probe:** During childbirth/labor and delivery?
        1. **Probe:** lack of client awareness, costs involved, geographical barriers
      - **Probe:** Postpartum?
        1. **Probe:** lack of client awareness, costs involved, geographical barriers
2. **From your perspective, what are some of the key reasons MassHealth-eligible people want doula care?**
   1. **Probe:** What are some of the barriers facing clients who seek doula care:
      - **Probe:** Prenatally?
        1. **Probe:** lack of client awareness, costs involved, geographical barriers
      - **Probe:** During childbirth/labor and delivery?
        1. **Probe:** lack of client awareness, costs involved, geographical barriers
      - **Probe:** Postpartum?
        1. **Probe:** lack of client awareness, costs involved, geographical barriers
3. **Much attention is being paid to racial and ethnic inequities in maternal health. What role, if any, does the doula play in addressing disparities?** *Your answer to this question may be similar to the one previously given responding to ways to address inequities in maternal health care.*
4. **[If applicable]:What are your thoughts about timing of enrollment in MassHealth and its relationship to MassHealth coverage of the doula benefit?**
   1. **Probe:** To your knowledge, what proportion of MassHealth-eligible individuals enroll in coverage at some point during the perinatal period?
   2. **Probe:** To your knowledge, when during the perinatal period do MassHealth-eligible individuals typically enroll in coverage?
5. **[If applicable]: To your knowledge, what proportion of MassHealth-insured individuals access doula services?**
   1. **Probe:** Prenatally?
      - **Probe:** When?
   2. **Probe:** During labor and delivery (i.e., once admitted to the hospital (for hospital deliveries))?
   3. **Probe:** Postpartum?
      - 1. **Probe:** When?
6. **[If time]: Please describe the state-level push for MassHealth coverage of doula care in MA?**
   1. **Probe:** Legislative push (i.e., H. 2372/S. 1475)?
      - **Probe:** Historical?
      - **Probe:** Current?
   2. **Probe:** Non-legislative push (i.e., coverage by MassHealth directly)?
      - **Probe:** Recent Request for Information (RFI)?
      - **Probe:** DPH Doula Coalition?
      - **Probe:** MA Doula Coalition Community Doula Workgroup?
7. **[If time]: How is planning for increased access to doula care in MassHealth configured in MA?**
   1. **Probe:** Please speak to issues related to doulas’ scope of services.
   2. **Probe:** “ “ training and core competencies.
   3. **Probe:** “ “ licensure and certification.
   4. **Probe:** “ “ relationship to clinical providers.
   5. **Probe:** “ “ process of compensation and reimbursement process.
   6. **Probe:** “ “ quality assurance and supervision.
8. **How will MassHealth-eligible individuals come to access doula care?**
   1. **Probe:**
      - Prenatally?
      - During labor and delivery (i.e., once admitted to the hospital (for hospital deliveries))?
      - Postpartum?
   2. **Probe:** How will MassHealth-eligible individuals become aware of doula services?
   3. **Probe:** How can MassHealth-eligible individuals best be informed about and gain access to doula care?
      - **Probe:** Please describe outreach and education efforts (if applicable).
9. **What are some of the barriers to establishing MassHealth coverage of doula care?**
   1. **Probe:** [See all probes related to question above.]
   2. **Probe:** Who are the most relevant players in establishing a public insurance coverage option for doula services?
10. **How do you feel about MA’s policy to increase access to doula care for MassHealth members (i.e., from your perspective, what value do doulas bring)?**

**THE FUTURE OF DOULA CARE.**

**1. In your opinion, what do you think should be done, if anything, at the national level with respect to supporting access to doula care?**

**CLOSING.**

- - - 1. **Would you like to share anything else that we have not covered during this interview?**

Those are all the questions I have for you at this time. Thank you again for your participation.

**[Stop recording]**
