# Supplementary material for: Access to perinatal doula services in Medicaid: a case analysis of 2 states
Source: Health Aff Sch. 2024 Mar 4;2(3):qxae023. doi: 10.1093/haschl/qxae023 (PMC10986220; doi:10.1093/haschl/qxae023)
Supplement: qxae023_Supplementary_Data [file qxae023_supplementary_data.zip › Appendix A4-codebook_R-R_1-3.pdf]

APPENDIX A4. CODEBOOK.

	Code	Description (presence or absence...)	Subcode(s) ["Child" code(s)]
ASPECTS OF POLICY			
	**Aspects of policy: Full-spectrum doula services**	All types of perinatal doula care	
	**Aspects of policy: Timing + # of doula care encounters**	E.g., reference to prenatal/postpartum visits	
CLIENT ISSUES			
	Client issue: access	Access to doula care for Medicaid members	Client issue: access - affordability
			Client issue: access - availability or
			Client issue: access - approachability
			Client issue: access - appropriateness
			Client issue: access - acceptability
	**Client issue: doula care utilization**	Issues related to utilization of doula care	
	**Client issue: payer outreach**	...by clinician or other healthcare personnel	
	**Client issue: provider outreach**	Outreach/marketing/advertising/education	
	**Client issue: referrals**	Referrals to doula care from provider	
	**Client issue: social determinants of health (SDOH)**	Necessity to address SDOH	
	**Client issue: word of mouth**	Clients access doula care via word of mouth	
COMPLEXITIES OF POLICY DEVELOPMENT			
	**Complexities of policy development: barrier to policy**	impeding sound policy	
	**Complexities of policy development: facilitator to**	enabling sound policy	
FUTURE DIRECTIONS: NATIONAL-LEVEL			
	**National-level policy efforts: collaboration**	...needed to offer blueprints for states	
	**National-level policy efforts: invest in research**	Invest in research nationally to look at outcomes associated with doula care utilization	
	**National-level policy efforts: health system capacity building**	Building capacity building of health system to equip it with doulas	
	**National-level policy efforts: standardization**	Necessity for standardization among doulas re: training/licensure/certification/credentialing/diversity	
PATHWAYS TO MEDICAID ENROLLMENT			
	**Pathways to enrollment: client**	Enrollment pre-conception<br><br>Enrollment due to pregnancy<br><br>Enrollment postpartum<br><br>(Note "unique nuance in MA: people who become pregnant are typically already enrolled in Medicaid")	
	**Pathways to enrollment: doula**	SPA, federal grants, inclusion in MMCO benefits plan, or other	
PAYER ISSUES			
	**Payer issue: reimbursement**	Reimbursement as a payer (i.e., Medicaid) issue	
	**Payer issue: ROI**	ROI as important to payers	
TENSION BETWEEN AGENCIES			
	**Tension between agencies: doula-executive**	Conflict among stakeholders	
	**Tension between agencies: doula-doula**	Conflict among stakeholders	

	**Tension between agencies: doula-legislative**	Conflict among stakeholders	
	**Tension between agencies: doula-provider**	Conflict among stakeholders	
	**Tension between agencies: executive-executive**	Conflict among stakeholders	
	**Tension between agencies: executive-legislative**	Conflict among stakeholders	
THOUGHTS ON POLICY			
	**Thoughts on policy: Conflicted feelings re: policy due to role in policy development or roles having changed**	Tension between informants' roles as state gov. rep. and maternal health care advocate or roles having changed	
	**Thoughts on policy: Confusion re: ongoing policy efforts**	Confusion re: doula care policy	
	**Thoughts on policy: Doulas as an opportunity for accountability in health care settings**	Doulas may provide some accountability to healthcare professionals (e.g., witnesses to obstetric violence or racism)	
	**Thoughts on policy: Perspective shift**	Description of perspective having changed about a policy	
	**Thoughts on policy: Proud of policy**	Proud of state policy	
	**Thoughts on policy: Progressive [local] culture affects policy**	Progressive state extends to health care and then to doula care/Medicaid policy	
	**Thoughts on policy: Doula hesitance re: policy**	Hesitance, distrust, or mistrust among doulas re: government or policy (for any reason)	
VIEWS OF OTHER STATES' DOULA POLICIES			
	**Negative views of other states' doula policies**	Negative perceptions of other states' doula care policies	
	**Positive views of other states' doula policies**	Positive perceptions of other states' doula care policies	
WORKFORCE ISSUES			
	**Workforce issue: background checks**	Necessary background checks prior to certification/registration	
	**Workforce issue: billing for services**	Discussion of doulas' needing to bill for their services; can include references to doula "hubs" (OR) or the DSPA (MA)	
	**Workforce issue: certification**	Certification as contentious policy point for doula workforce; can include allusions to licensure or credentialing	
	**Workforce issue: diversity/cultural concordance**	Diversity/coula-client cultural concordance and/or racial equity as contentious policy point or consideration for doula workforce	
	**Workforce issue: doula-Medicaid interactions**	Challenges for doulas re: interacting with Medicaid (i.e., getting paid/reimbursed)	"no apparent reasons"<br>"glitch in the system"<br>payment timeliness
	**Workforce issue: enrollment as a Medicaid provider**	Administrative or bureaucratic obstacles facing doulas when enrolling (e.g., paperwork burden)	
	**Workforce issue: health system integration**	Integration of doulas into healthcare system	

	**Workforce issue: compensation/reimbursement**	Reimbursement as a workforce issue (e.g., leading to no incentive/motivation to enter or stay in workforce; leads to high attrition); may or may not refer to specific reimbursement rate(s)	randomness of late/lack of research or pre-work<br><br>seeking input from other states' policies<br><br>can refer to voluntary nature of policy/doulas' ability to opt-in to reimbursement
	**Workforce issue: scope of practice**	Activities performed as as permitted or defined (or not) within the doula profession	
	**Workforce issue: professional development**	Opportunities or lack thereof for professional development for the doula workforce, including workforce expansion, doula workforce advocacy and supervision/surveillance/mentorship	
	**Workforce issue: training**	Training as contentious policy point for doula workforce; can also include discussion of achievement of core competencies	
**INTERESTING QUOTE**			
	**Interesting quote**	e.g., OR-3:<br><br>"one fell swoop"<br><br>"low-hanging fruit"<br><br>"incentive"<br><br>"motivation" (re: reimbursement rate)<br><br>"win win win"	
**MISCELLANEOUS**			
	**Doula diversity/doula-client cultural concordance**	Diversity/doula-client cultural concordance and/or racial equity as a policy point or consideration for doula workforce	
	**"Getting it right"**	Description of building an imperfect policy and refining it afterward versus taking the time and resources to "get it right" from the getgo without having to do "cleanup" (Amy Chen) (i.e., revise the policy) - afterward	
	**Novelty of doula care as a concept**	Doula care is novel to many parties	
	**Power/influence**	Power or influence of some institutions over others; may or may not result in TENSION between parties/agencies; these can include the doula workforce	
	**Uncertainty re: timing of enrollment in Medicaid**	Participant doubt re: process of client enrollment in Medicaid and receiving benefits	
	**Medicaid: Golden Ticket**	Description of Medicaid reimbursement for doula services serving as a golden ticket, silver bullet, or panacea, in that it will or will not solve all problems related to maternal health, etc.; could also include mention of advocacy for alternate options to Medicaid reimbursement	
	**COVID**	Consequences/repercussions of pandemic on provision of doula services [in Medicaid]	
