# Supplementary material for: Access to perinatal doula services in Medicaid: a case analysis of 2 states
Source: Health Aff Sch. 2024 Mar 4;2(3):qxae023. doi: 10.1093/haschl/qxae023 (PMC10986220; doi:10.1093/haschl/qxae023)
Supplement: qxae023_Supplementary_Data [file qxae023_supplementary_data.zip › Appendix A5_Supplemental Material_EPIS.docx]

| **EPIS Constructs** | **EPIS Domains** | **Oregon (Sustainment)** | **Massachusetts (Preparation)** | **Examples** |
| --- | --- | --- | --- | --- |
| **Outer context** | **Leadership** |  |  | CMS; state legislature |
|  | **Inter-organizational environment** |  |  | Medicaid structure: MassHealth/ACOs or MCOs; billing and reimbursement process |
|  |  |  |  | Medicaid structure/OHP/CCOs |
|  | **Client characteristics** |  |  | Medicaid members |
|  | **Service environment** |  |  | Stakeholders’ perceptions of doula care |
|  | **Sociopolitical & media attention** |  |  | US maternal health crisis |
| **Inner context** | **Leadership** |  |  | Medicaid leaders;  state government administrators; clinicians;  doulas |
|  | **Organizational characteristics** |  |  | Size and structure of Medicaid and other state agencies; healthcare institutions’ readiness for change (i.e., doulas) |
| **Bridging factors** | **Community-Academic [Governmental] partnerships** |  |  | THW liaisons, (contract with CCOs to provide billing and administrative support to doulas); groups like Oregon Doula Association; Oregon Doulas of Color |
|  |  |  |  | Community Doula Coalition; Interagency Meetings; MassHealth Public Listening Sessions |
| **Innovation factors** | **Innovation characteristics** |  |  | Medicaid reimbursement rate, training and education requirements; credentialing system(s); number, frequency, and timing of prenatal + postpartum visits reimbursable |
|  |  |  |  | Reimbursement rate increase; number, frequency, and timing of prenatal + postpartum visits reimbursable |
